# A changing landscape in gastric cancer

**DOI:** 10.3322/caac.70113

**Published:** 2026-09-30

**Authors:** Lindsay M. Hannan, Vaia Florou

**Affiliations:** ^1^ Department of Hematology and Medical Oncology Winship Cancer Institute of Emory University Atlanta Georgia USA; ^2^ Division of Oncology, Department of Internal Medicine University of Utah Health Care Huntsman Cancer Institute Salt Lake City Utah USA

## Abstract

New treatments are improving outcomes for patients with gastric cancer. Determining how to best select, combine, and sequence these therapies remains a clinical challenge.

Gastric cancer (GC) remains one of the most lethal cancers globally. In this issue of *CA: A Cancer Journal for Clinicians*, Wang et al. provide a comprehensive review of recent advances across the spectrum of GC.^1^ The rapidly evolving treatment paradigm of metastatic disease is particularly striking and is the focus of this editorial. Clinicians now face questions that were uncommon a decade ago: Which treatment should take priority when several biomarkers are actionable, and how should that choice influence subsequent therapy? The emergence of claudin 18.2 (CLDN18.2)‐directed antibodies, antibody–drug conjugates, and cellular therapy makes these questions increasingly relevant.

For many years, palliative systemic treatment relied almost entirely on cytotoxic chemotherapy along with trastuzumab as an option for human epidermal growth factor receptor 2 (HER2)‐positive disease^2^ and the vascular endothelial growth factor 2 (VEGFR2) antagonist ramucirumab as an option after first‐line therapy.^3^ Only experimental options were available once these therapies became ineffective or intolerable. This landscape has changed considerably over the past 5 years; Biomarker‐driven treatment is now part of routine clinical practice, and actionable targets continue to evolve. The addition of immune checkpoint inhibitors to chemotherapy was one of the recent significant advancements.^4^, ^5^, ^6^ Programmed death ligand‐1 (PD‐L1) expression, quantified using the combined positive score (CPS), is used to identify patients most likely to benefit from immunotherapy, with the magnitude of benefit generally increasing with higher CPS. Treatment decisions remain difficult for tumors with low PD‐L1 expression, in which the incremental benefit is less clear. Microsatellite instability‐high status and, in selected settings, high tumor mutational burden provide additional information regarding the suitability of treatment with immune checkpoint inhibitors, but this pertains to only a relatively small proportion of patients.

HER2‐directed therapy has also expanded. HER2 is overexpressed in a subset of GCs^7^ and has been successfully targeted with trastuzumab, the antibody–drug conjugate fam‐trastuzumab deruxtecan‐nxki, and the dual HER2‐targeted, bispecific antibody zanidatamab. The eligibility criteria for first‐line combinations differ. Pembrolizumab with trastuzumab and fluoropyrimidine‐containing and platinum‐containing chemotherapy is approved by the US Food and Drug Administration (FDA) for first‐line treatment of HER2‐positive advanced gastric or gastroesophageal junction adenocarcinoma with a PD‐L1 CPS ≥1.^5^ However, the combination of zanidatamab, tislelizumab, and chemotherapy, which was approved by the FDA on August 25, 2026, does not require PD‐L1 positivity.^6^ These distinctions matter when using biomarker results to select a regimen.

CLDN18.2 has emerged as another important target,^8^ with the anti‐CLDN18.2 monoclonal antibody zolbetuximab approved for first‐line treatment of HER2‐negative, CLDN18.2‐positive, advanced disease in combination with fluoropyrimidine‐containing and platinum‐containing chemotherapy.^9^ These advances raise an increasingly important clinical question: What should we do when more than one biomarker is actionable? This question is particularly relevant for patients whose tumors express CLDN18.2 and have a high PD‐L1 CPS. Both immunotherapy and CLDN18.2‐directed treatment may be reasonable options. Still, we have limited evidence to date to guide us on which should take priority, whether they should be combined, or how they should be sequenced. Recent studies have begun to address the combination question. In cohort 4 of the phase 2 ILUSTRO trial,^10^ patients with HER2‐negative, CLDN18.2‐positive disease received zolbetuximab with nivolumab and modified leucovorin (folinic acid), fluorouracil, and oxaliplatin (mFOLFOX6). Both PD‐L1–positive and PD‐L1–negative tumors were included. The efficacy and safety findings support further evaluation of this combination, but the absence of a randomized comparator precludes conclusions about the contribution of each agent or its advantage over existing standard‐of‐care regimens. The currently accruing phase 3 LUCERNA trial aims to evaluate the efficacy of adding zolbetuximab to chemoimmunotherapy in patients with HER2‐negative, CLDN18.2‐positive, PD‐L1–positive disease, with 1:1 randomization of treatment‐naive patients to the experimental arm of mFOLFOX or CAPOX (capecitabine plus oxaliplatin) plus pembrolizumab plus zolbetuximab or to the control arm of mFOLFOX or CAPOX plus pembrolizumab plus placebo.^11^ The trial should clarify the incremental benefit of adding zolbetuximab to chemoimmunotherapy, although it will not establish the optimal treatment sequence.

A different combination is being evaluated in the CLARITY GASTRIC‐02 study (ClinicalTrials.gov identifier NCT07431281), which evaluates rilvegostomig, an anti–PD‐1 and anti‐TIGIT bispecific antibody, and sonesitatug vedotin, an antibody–drug conjugate against CLDN18.2, with capecitabine in cohort 1 patients with CLDN18.2‐positive and PD‐L1–positive gastroesophageal cancers. As these approaches develop, treatment selection will require evidence specific to the agent and combination being considered.

Perhaps the most consequential recent development, however, is the emergence of cellular therapy. Chimeric antigen receptor (CAR) T‐cell (CAR‐T) therapy has transformed the treatment of hematologic malignancies, but obtaining similar success in solid tumors has been difficult. The approval in China of satricabtagene autoleucel (satri‐cel), a CLDN18.2‐directed therapy, marks the first CAR‐T approval for a solid tumor^12^ and suggests that some of the barriers limiting its efficacy in solid tumors may be overcome. In patients with previously treated, HER2‐negative, CLDN18.2‐positive, advanced GC, satri‐cel demonstrated improved clinical outcomes compared with physician’s choice treatment.^13^ The significance of this development extends beyond the efficacy of an individual agent. It adds a new treatment dimension to GC, making the integration of CAR‐T therapy with existing treatments a practical clinical question.

Decisions regarding optimal regimen selection and sequence become more complicated as additional agents and cellular therapies enter clinical practice. In addition to efficacy, toxicity is also an important feature in regimen selection. Nausea and vomiting^14^ can be substantial with CLDN18.2‐directed antibody therapy, whereas CAR‐T therapy brings a different set of side effects, including cytokine release syndrome and other immune‐mediated toxicities.^13^ Thus treatment selection will increasingly require consideration of the therapeutic target, treatment modality, prior therapy, treatment‐related toxicity, financial burden, and patient characteristics, alongside biomarker rather than the presence or absence of a biomarker alone.

Durability of response and resistance remain important challenges. Antigen loss and downregulation are well recognized mechanisms of resistance to cellular therapy, and the suppressive microenvironment of solid tumors may limit the efficacy of cell therapies.^15^ Approaches under investigation, including dual‐target CARs, strategies to improve T‐cell persistence, and combinations with checkpoint inhibitors, may help address these limitations. Their clinical value will depend on whether they extend disease control without an unacceptable increase in toxicity. Understanding resistance after each treatment may also help determine whether another agent directed at the same target remains a reasonable option.

A fundamental limitation is that single biomarkers at diagnosis may not remain representative throughout treatment. GC is heterogeneous, both between patients and within an individual tumor, and its molecular profile can evolve with treatment. A single biopsy obtained at diagnosis may provide an incomplete picture of the disease later in its course. Incorporating serial molecular profiling, circulating tumor DNA, and other multi‐omic approaches may enable treatment decisions to reflect how the tumor changes over time rather than relying on a static biomarker result.

Wang et al. describe a field that has changed substantially. The question is no longer simply whether a patient with metastatic GC has an actionable biomarker. Increasingly, the challenge will be deciding which biomarker matters most at a given point in the disease course and how to combine or sequence the available therapies. For CLDN18.2‐directed therapies, this includes whether treatment with an antibody or antibody–drug conjugate affects subsequent benefit from cellular therapy. Trials should address treatment outcomes, sequencing, and quality of life. Whether CAR‐T therapy will ultimately become part of routine treatment for GC worldwide remains to be determined, but its first approval for a solid tumor makes planning for its place in the treatment sequence particularly timely.

CONFLICT OF INTEREST STATEMENT

Lindsey M. Hannan reports grants/contracts from AstraZeneca, Coherus Biosciences Inc., and Johnson & Johnson outside the submitted work. Vaia Florou worked as consulting or advisory: Aadi Bioscience, Deciphera, SpringWorks Therapeutics, Merus, Ipsen, Exelixis.
